# Uterine cervix cancer treatment at Radiumhemmet: 90 years′ experience. Time trends of age, stage, and histopathology distribution

**DOI:** 10.1002/cam4.187

**Published:** 2014-01-14

**Authors:** Kristina Hellman, Ann-Cathrin Hellström, B Folke Pettersson

**Affiliations:** Department of Gynecological Oncology, Radiumhemmet, Solna, Karolinska Institutet, Karolinska University HospitalStockholm, Sweden

**Keywords:** Age, cervical carcinoma, histopathology, history, stage

## Abstract

Since the introduction of screening programs for cervical cancer (CC) the incidence has decreased and CC is discovered at an earlier stage. The purpose of this study was to analyze time trends in age, stage, and histopathology over a 90-year period and to our knowledge this is the largest single institutional series in the literature of invasive cervical carcinoma (CC) cases. This is a retrospective study comprising 18,472 women treated for CC from 1914 until 2004 at Radiumhemmet, Stockholm. The material is part of the international CC statistics published since 1937 in the League of Nations' Annual Reports, and since 1958 under the patronage of International Federation of Gynecology and Obstetrics (FIGO). During the 90-year study period, the annual number of cases treated increased to over 400 up until 1965, after which there was a gradual drop to less than 100 cases in 2004. A pronounced shift toward earlier stages at diagnosis was noted. The mean age at diagnosis increased in all stages, predominantly in advanced stages. A reduction in squamous cell carcinoma (SCC) cases and a sixfold increase in the proportion of adenocarcinoma (AC) cases were observed. The mean age at diagnosis for squamous and AC cases shifted after 1970, when the SCC cases ultimately became 3 years older than the AC cases in contrast to around 1950 when they were 3 years younger than the AC cases. The changes in the distribution by age, stage, and histopathology during this 90-year period are probably associated with: improved social conditions and increased access to health care, the introduction of screening programs for CC in the 1960s, and a change in the risk factors for CC (changed sexual behavior, introduction of contraceptive pills, and changed smoking habits).

This is a study on changes in the distribution by age, stage, and histopathology in a large series of cervical cancer treated in Sweden during a 90-year period. It also includes an historical review about the development of staging rules and the international reporting system for gynecological cancers.

## Introduction

During the last century, knowledge about the carcinogenesis and etiology of cervical carcinoma has increased immensely. Infection with oncogenic types of human papillomavirus (HPV) has been identified as the major cause of cervical cancer (CC) 1–4.

**Figure 1:**
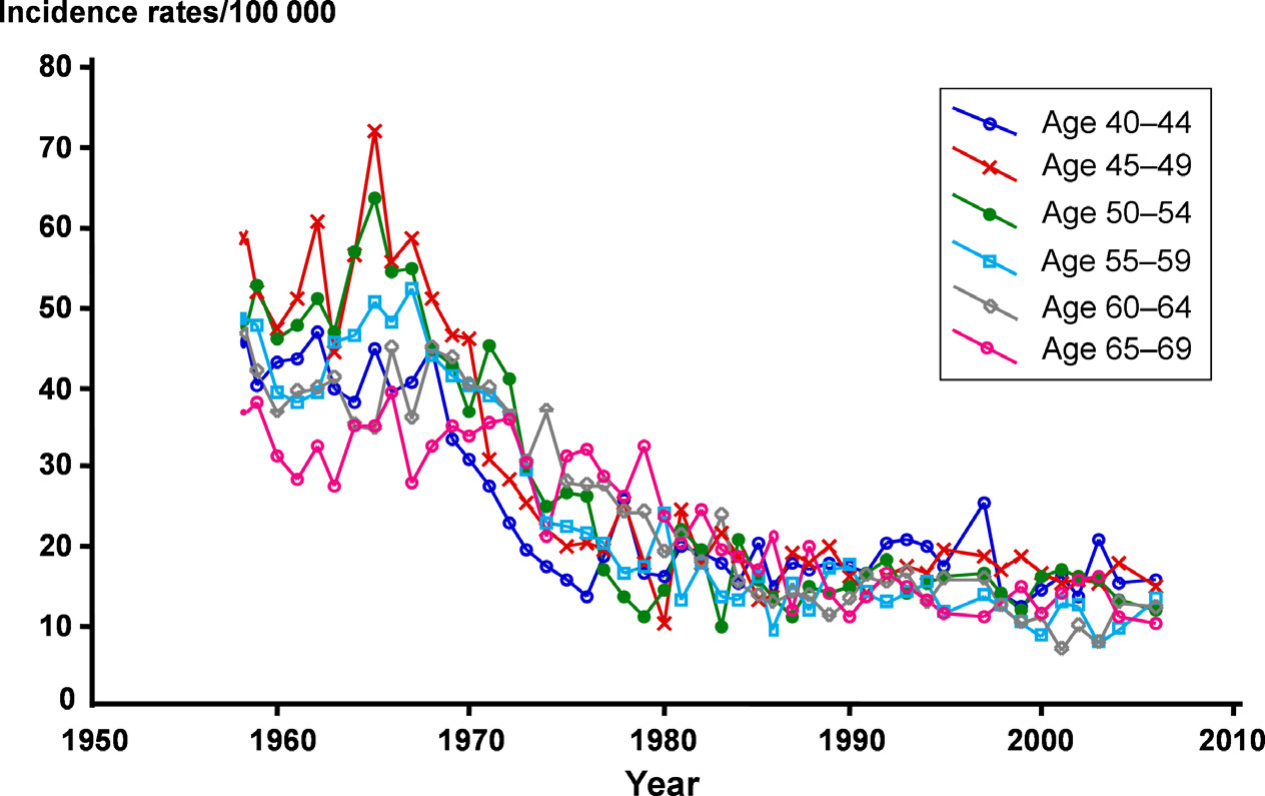
Carcinoma of the uterine cervix age-specific incidence rates/100,000 in Sweden in age groups from 35–39 to 60–64. Data from the National Swedish Cancer Registry.

**Figure 2:**
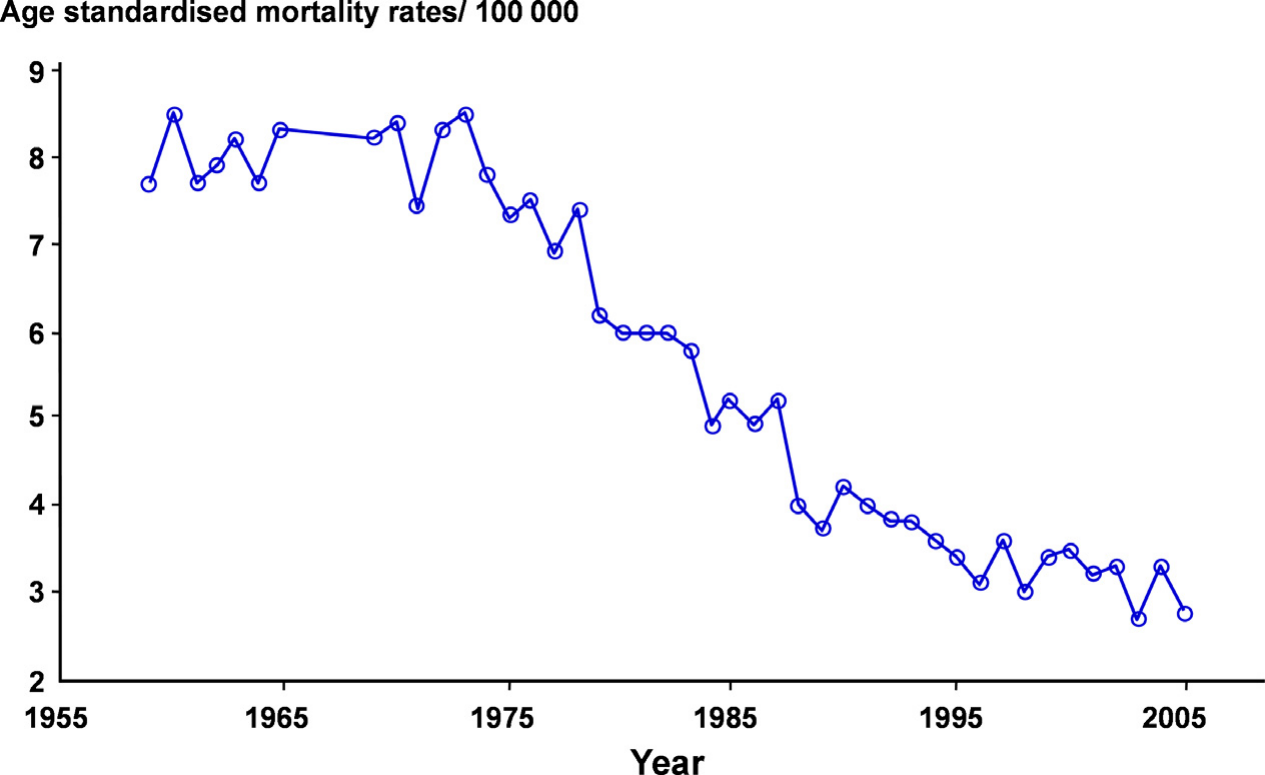
Age standardized mortality rates for carcinoma of the uterine cervix in Sweden per 100,000 women. The Swedish Cause of Death Register.

In Sweden, there has been a declining incidence rate and a reduced mortality rate for CC in recent decades (Figs. 1 and 2). The lowest incidence rates are found in Europe (excluding some eastern European countries), North America, and China 4. Before the introduction of screening programs the incidence was similar to that seen in many developing countries today.

According to the International Federation of Gynecology and Obstetrics (FIGO) Annual Reports on the results of treatment of gynaecological cancers the observed total survival rates for CC given as 5- or 10-year survival have improved during the last 90 years with the exemption for the most recent years 5. Changes in survival rates in CC may be due to a gradual change in the composition of the predicting variables of the series referred for treatment, that is, age, stage, and histopathology distribution. Some of these changes may be an effect of the screening activities 4. Studies have shown a change in the distribution by age, stage, and histopathology in the past decades; cervical carcinoma is detected at an earlier stage 4, the incidence of squamous cell carcinomas (SCCs) has declined, and the number of adenocarcinomas (ACs) has increased 6–8, there has also been a change in age at diagnosis between advanced-stage and early-stage CC 9.

In the early 1900s, some pioneer physicians in Sweden participated in the fundamental work that contributed to development of treatment techniques for CC, staging rules, and to building up an international reporting system (the Annual Reports on the results of treatment of gynecological cancers) (for more details see Material and Methods.

### Historical notes

Until the beginning of 19th century, the only treatment for cervical carcinoma was surgery, and the results were poor. The picture changed radically, however, following Marie and Pierre Curie's discovery of the potentiality of radium (1898). The American surgeon Robert Abbe (1851–1928) was the first to use radium in the treatment of cervical carcinoma (1910). Gösta Forsell (1876–1950) in Stockholm applied radium in inoperable cases of cervical carcinoma (1912) and reported clinical healing in several cases. Forsell presented the Stockholm method for treatment of cervical carcinoma in 1914. He soon realized the necessity of cooperating with surgeons trained in gynecological operations and centralizing the treatment to a few hospitals. James Heyman (1882–1956) was asked to cooperate and together with Forsell they further developed the Stockholm method of treatment. This was the start of Radiumhemmet. After a speech by James Heyman in Gothenborg (1927), it was decided that all cases of carcinoma of the cervix uteri in Sweden, irrespective of age or operability, should be referred for primary irradiation.

All CC cases treated at Radiumhemmet during a 90-year period, from 1914 until 2004 (18,472 cases), form the basis of this study. The aim of the study was to describe and analyze time trends in the distribution by age at diagnosis, stage, and histopathology that could have affected the incidence and survival rates.

## Materials and Methods

### Staging rules

**Figure 3:**
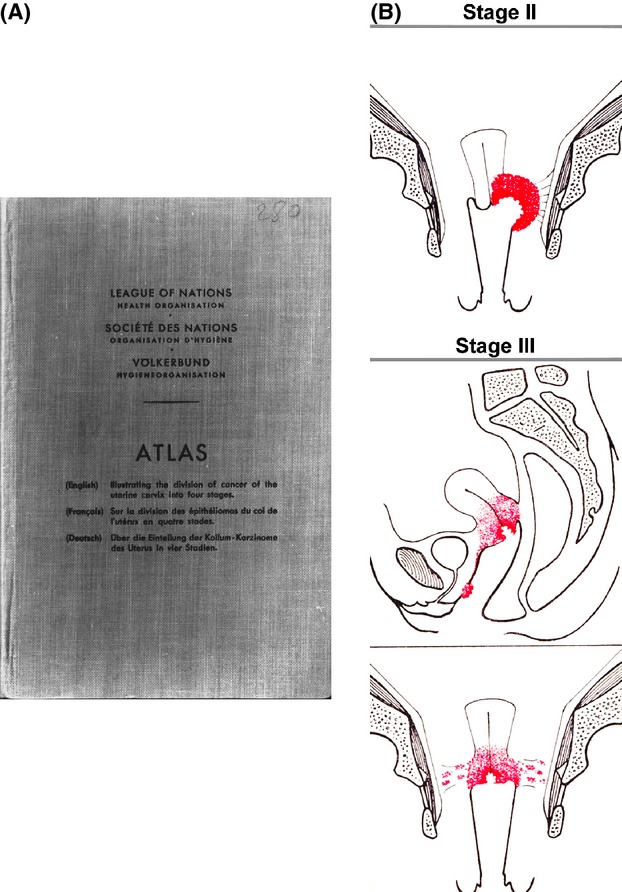
(A) The first Atlas for staging of cervical carcinoma was published by the League of Nations, 1929. (B) A system for grouping cervical cancers into four stages according to the extent of the disease was produced by the professors Heyman, Lacassagne and Voltz. The diagrams have been drawn by Dr. Strandquist.

In 1928, the Radiological Sub-Commission of the Cancer Commission of the Health Organization of the League of Nations was invited to report on the possibility of presenting uniform statistical statements on the results obtained by radiotherapeutic methods in the treatment of carcinoma of the uterine cervix. Thus, the Sub-Commission recognized that reliable information regarding the results obtained by different methods could be procured only if those results were recorded in a uniform way. The task of formulating rules designed to facilitate the presentation of exactly comparable statistics was entrusted to Dr. J. Heyman, Stockholm, Dr. A. Lacassagne, Paris, and Prof. F. Voltz, Munich. The experts' suggestions were adopted in a slightly modified form by the Sub-Commission, and published in April 1929. The recommendations were adopted by Radiumhemmet, and all cases were clinically staged under anesthesia before treatment. Experience has shown, however, that the rules were somewhat differently interpreted and that such variations in interpretation tended to defeat the effort to secure comparability in the statistics. Therefore, the Committee was entrusted with preparing of a volume illustrating, by appropriate diagrams, examples of the cases which should be allocated to each of the four stages. This proposal was adopted by the Health Organization, and work on the preparation of the Atlas was assigned to Dr. J. Heyman in collaboration with Dr. M. Strandquist, Stockholm 10 (Fig. 3A). The diagrams were drawn by Dr. Strandquist (Fig. 3B). Presentation of the volume provided the opportunity to slightly modify the definitions accepted in 1929; the proposed alteration of the definition for stage IV reduced the number of cases referred to that group.

### Annual reports

All cases treated at Radiumhemmet from 1914 until 1989 (vol. 22) were reported in the Annual Report. The Annual Report was annual for the initial years, but later data were collected every 3 years (from 1973). The League of Nations was responsible for the Report, but beginning in 1958 responsibility was assumed by FIGO. Starting from the first Report the editors were James Heyman (vol. 1–10), Hans Ludwig Kottmeier (vol. 11–18), and Folke Pettersson (vol. 19–22) at Radiumhemmet in Stockholm, all of whom took active part in the work of the international cancer committee, which was responsible for changes in the rules of staging.

The first Annual Report contained results from six institutions (Centre des Tumeurs de ′Úniversite′ de Bruxelles, Belgium, The Liverpool Radium Institute, England, The Marie Curie Hospital, London, England, The Radium Centre for Carcinoma of the Uterus, London County Council, England, Institut du Radium de l ′Université′ de Paris, France, and Radiumhemmet, Stockholm, Sweden). A total of 3222 cases of cervical carcinoma treated up until 1930 were reported to the Annual Report, out of which Radiumhemmet contributed 1591 cases treated during the years 1914–1930 (vol. 1).

A total of 5659 cases from Sweden were reported to the Annual Report from 1914 until 1941 (vol. 1–5) 11–15 of which 413 were reported from Lund, 854 from Gothenburg and 4392 from Radiumhemmet (77.6 percent).

The National Swedish Cancer Registry was started in 1958, and from 1958 to 1989 22,520 cases of cancer of the uterine cervix were reported, and of these 7283 cases (32.2%) were treated at Radiumhemmet and reported to the Annual Report. Thus, a significant proportion of the carcinoma of the uterine cervix cases treated in Sweden have been treated at Radiumhemmet; 77.6% until 1941 and 32.2% between 1958 and 1989.

### Material

In total, the records for the 90-year period from 1914 to 2004 comprise 18,472 women treated for invasive carcinoma of the uterine cervix at Radiumhemmet, and these results form the basis of this study. Initially patients were referred from all of Sweden, but gradually treatment centers were set up in other parts of the country including Gothenburg, Lund, Umeå, Uppsala, Linköping, and Örebro. In recent years, patients have been referred to Radiumhemmet from hospitals within the Stockholm-Gotland region with a population of 1.8 million.

For the 22nd Annual Report published in 1995, 104 collaborating institutions from all over the world contributed 22,262 individual cases with data on age, stage, histopathological type, treatment modality, and follow-up data, and Radiumhemmet contributed 315 of these cases 16.

Follow-up was continued until the end of life and the register is complete apart from a small, inevitable loss to follow-up due to emigration. Most patients were followed through personal visits and annual examinations at Radiumhemmet. In some cases, follow-up was continued through correspondence with the patients and their local doctors. Death certificates were obtained from parish priests and in later years (starting in 1958) through the Swedish Cause of Death Register.

### Classification of histopathological classes

All old microscopic specimens from the tumors were re-evaluated by Radiumhemmet pathologists and if necessary new specimens were taken before treatment. This procedure could be performed when the patients had been referred to the clinic and when they went through the pretreatment evaluation and staging that were thoroughly performed under general anesthesia.

Only invasive cancers were included in the study and the histological evaluation was done according to the World Health Organization classification 1975, no. 13.

### Statistical analysis

The statistical analysis was made using Statistica version 4.1. In this large descriptive retrospective material, mean values have been chosen to show changes over time. This has been presented in figures or tables. No statistical comparisons have been performed.

## Results

**Figure 4:**
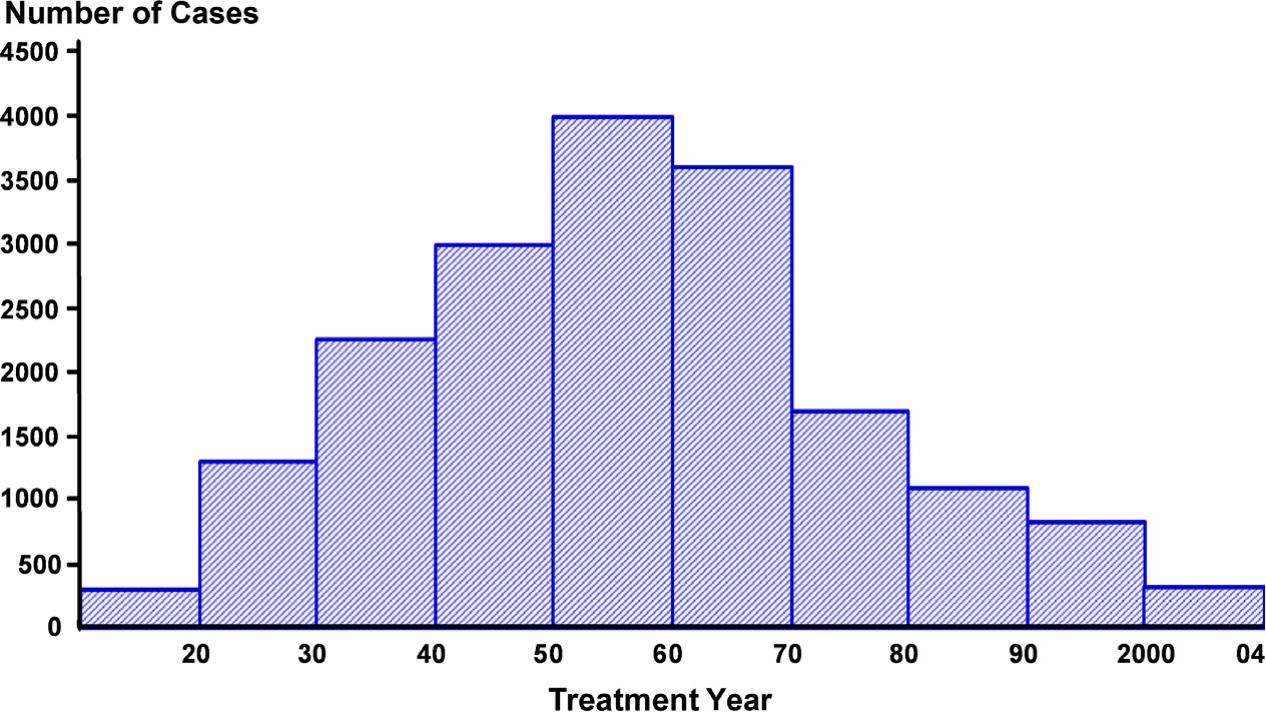
Carcinoma of the uterine cervix treated at Radiumhemmet 1914–2004 (*n* = 18,472).

The annual numbers of CC cases treated at Radiumhemmet between 1914 and 2004 are shown in Figure 4. In 1914, fewer than 50 cases were treated. A gradual increase resulted in 400 cases annually from 1950 to 1965 and thereafter a slope formed drop to fewer than 100 cases yearly from 1990 to 2004. A significant proportion of the CC cases treated in Sweden has been treated at Radiumhemmet; 77.6% until 1941 and 32.2% from 1958 to 1989. In recent years approximately one quarter of the CC cases in Sweden have been treated at Radiumhemmet.

**Figure 5:**
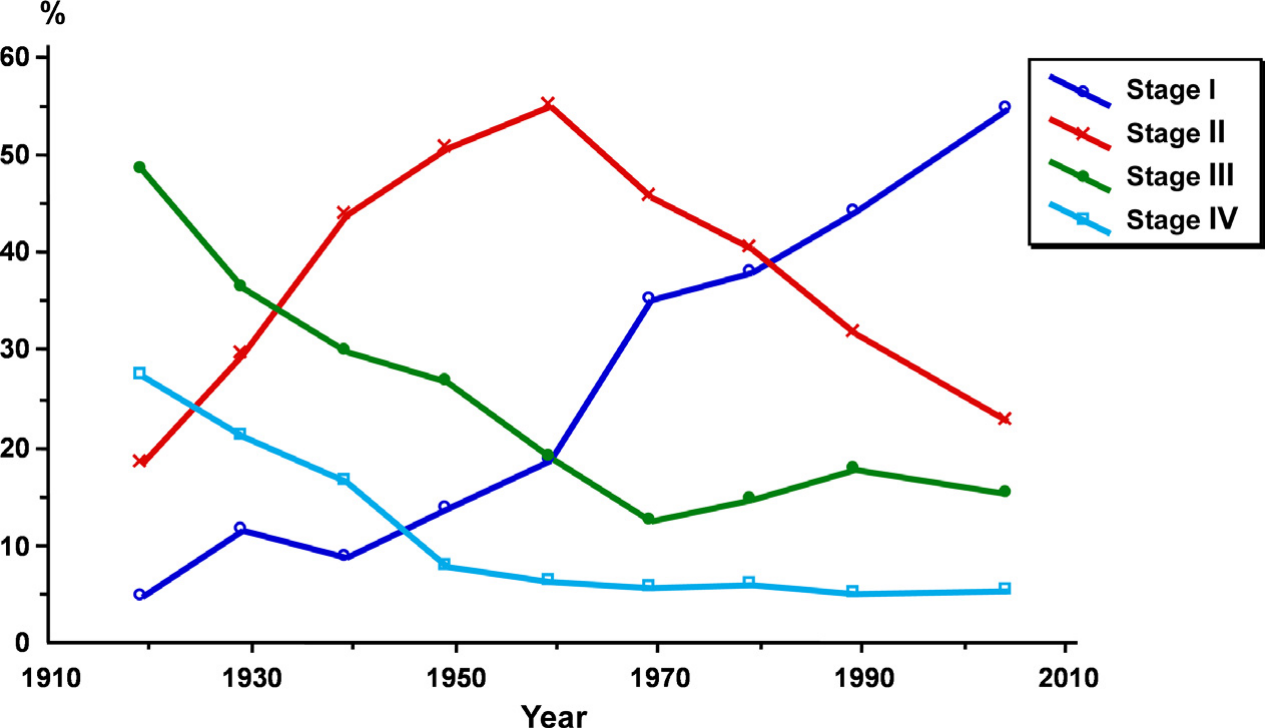
Distribution of stage by year of treatment of 18,472 cases of carcinoma of the uterine cervix treated at Radiumhemmet.

The stage distribution shows a continuous shift toward earlier stages at diagnosis during the study period (Fig. 5). Starting as early as 1940, the percentage of stage I cases increased and this has been even more pronounced since 1960. The percentage of cases in stages III and IV decreased in 1930, but after 1960 the drop has leveled out. Stage II cases increased from 1920 to 1959 and thereafter decreased.

**Figure 6:**
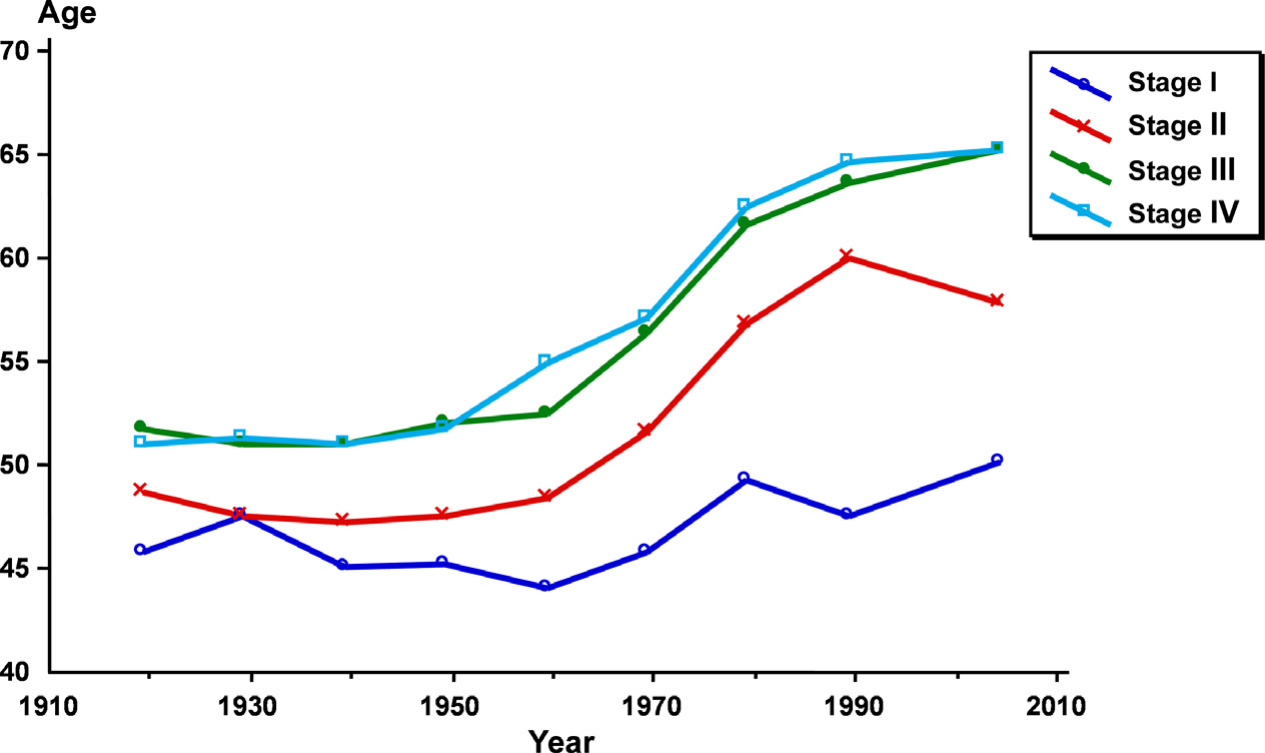
Age at diagnosis by treatment period and stage (*n* = 18,472).

The mean age at diagnosis decreased moderately for stage I cases from 1930 until 1959, when there was a slight increase (Fig. 6, Table 1). For stages II–IV an obvious increase in mean age is seen after 1960. For stage I, the mean age at diagnosis increased by 4.3 years (8.6%) from 1919 to 2004 (from 45.9 to 50.2 years). For stage IV, the mean age increased by 14.3 years (21.8%) during the same period (from 51.1 to 65.4 years) (Table 1).

**Table 1:** Cervical carcinoma

Period of treatment	Stage I	Stage II	Stage III	Stage IV
1914–1919	45.9	48.8	51.9	51.1
1920–1929	47.7	47.7	51.1	51.5
1930–1939	45.2	47.4	51.2	51.1
1940–1949	45.3	47.7	52.2	51.8
1950–1959	44.2	48.5	52.6	55.1
1960–1969	45.9	51.7	56.5	57.2
1970–1979	49.4	56.9	61.8	62.6
1980–1989	47.6	60.2	63.8	64.8
1990–2004	50.2	58.0	65.4	65.4

Age at diagnosis by treatment period and stage (*N* = 18,472).

**Figure 7:**
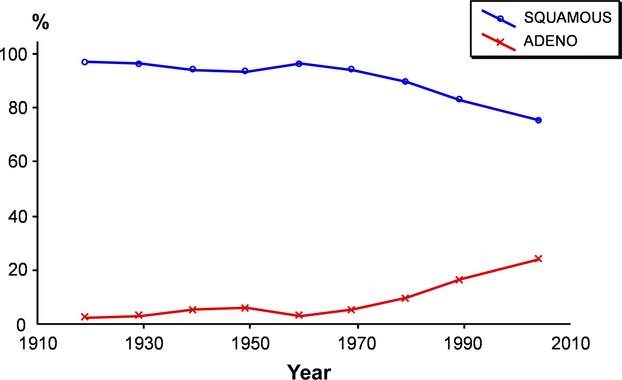
Distribution of histopathology by year at treatment (*n* = 18,472).

The proportion of SCC cases was reduced at the expense of the AC cases, which increased from about 4% to around 26% (Fig. 7 and Table 2). The main change was noted after 1960.

**Table 2:** Cervical carcinoma

Period of treatment	Squamous cell carcinoma (%)	Adenocarcinoma (%)
1914–1919	96.6	3.4
1920–1929	95.6	4.4
1930–1939	93.3	6.7
1940–1949	92.5	7.5
1950–1959	95.7	4.3
1960–1969	93.1	6.9
1970–1979	89.0	11.0
1980–1989	82.3	17.7
1990–2004	74.3	25.7

Distribution of histopathology by year at treatment (*N* = 18,472).

**Figure 8:**
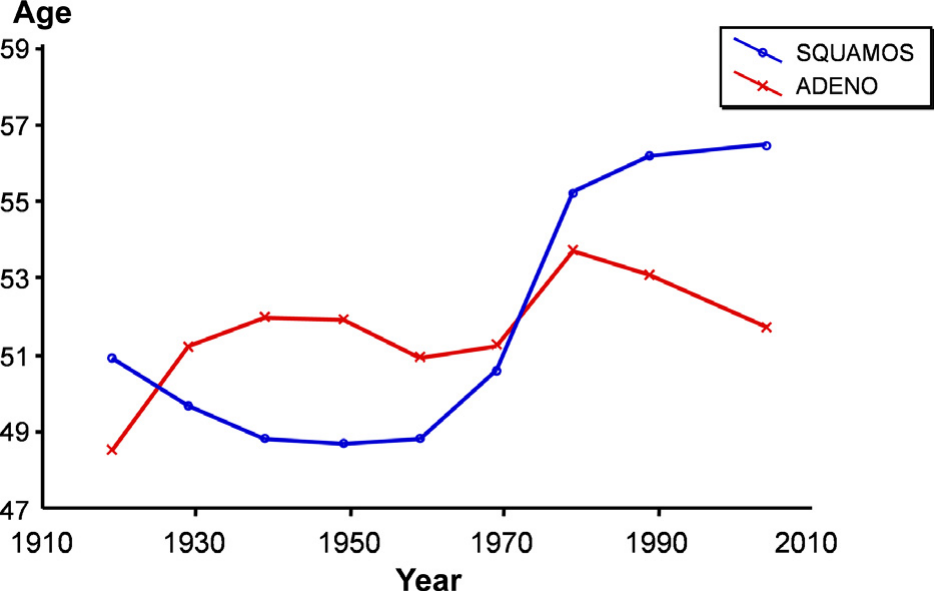
The mean age at diagnosis for adenocarcinoma and squamous cell carcinoma (*n* = 18,472).

The mean age at diagnosis for AC and SCC in all stages is shown in Figure 8. With the exception of the years before 1930, with fairly small materials, especially for the AC cases, the SCC cases were 2–3 years younger than the AC cases. After 1960 a shift is seen, with an increase in mean age for SCC cases to more than 3 years older than the AC cases.

## Discussion

This study is based on the material comprised of cervical carcinoma cases (18,472 cases) treated during a 90-year period at the Department of Gynecological Oncology at Radiumhemmet, Karolinska University Hospital in Stockholm (1914–2004).

It is seen that the variables studied have undergone a gradual change during the time period. Similar changes of the variables are described by the different collaborating institutions in the Annual Reports 11–15. There has been a continuous shift toward earlier stages at diagnosis. The proportion of stage I cases increased from the beginning of the study period, most likely due to changes in the health care system, and after 1960 probably due to an effect of organized screening. The percentage of stage II cases increased rapidly in the pre-screening period and thereafter declined. The percentage of advanced stages dropped early, before the screening period, and thereafter remained quite unchanged at a low level. The recent picture is dominated by stage I, comprising more than 50% of all cases.

The mean age at diagnosis decreased for stage I cases from 1930 until 1959 and thereafter a slight increase occurred. For stages II–IV an obvious increase in mean age is seen as early as after 1950. With a stable population and with no environmental effects acting on that population, the natural history of the disease would certainly have created a stable picture with stable intervals between the different stages and the different periods. And this should also have resulted in a stable situation with regard to the age for the stages in the different periods, which is not seen. On the contrary, the material is continuously changing, probably due to the impact of development of the health care system, education of the population, changing sexual behavior, use of new contraceptive methods, use of estrogens, changing carcinogens, cigarette smoking and ultimately, and not of least importance the effect of the organized screening.

The proportion of SCC cases was reduced at the expense of the AC cases, which increased approximately sixfold with the main change noted after 1960. In the present material, the SCC cases are reduced to 75%, while the AC cases are increased to 25% (six times more). In real numbers, this means around 75 cases of SCC and around 25 cases of AC are diagnosed each year in the Stockholm-Gotland region.

Data from the Swedish National Cancer Registry for the period 1958–1980 show a successive relative increase in the percentage of AC cases among all cervical carcinomas counted by births cohorts and age groups. However, the true incidence rate of AC cases, with the inclusion of the cases of mucoepidermoid cancer of the uterine cervix, was practically unchanged over birth cohorts and age groups during this period. An increase was seen only in the younger age groups: from 0.7 to 1.2 per 100,000 women in the age group 25–29 years and from 1.0 to 1.8 in the age group 30–34 years. This should contribute to an increase in AC cases after 1965 17,18. Those younger girls had most probably been exposed to new or changed risk factors. However, the most important reason for the increase in AC cases is dependent on a selective process in the screening programs where the SCC cases are easier to detect than the AC cases.

In contrast to SCC cases, which undergo a pronounced increase in age at diagnosis, the AC cases show only a moderate increase during the period under study.

During this 90-year period in Sweden the structure of society, the population, and health care services have undergone immense development and change that have most likely had an impact on the variables studied. In the 18th century, Sweden was dependent primarily on agriculture with only about 7% of the population living in cities. During the 19th century, Sweden developed into an industrial country, and by 2005 the population had almost doubled, and 85% of the inhabitants were living in cities 19. In parallel with this urbanization, knowledge in medical science progressed rapidly and health care and medical services were developed and became available to the whole population. During the period of one century, up until 2000, the number of medical doctors increased from 1000 to 28,000. Development of the different medical specialties occurred in parallel. The “diseases of the women” specialty was noted as early as 1915. Thereafter gynecological radiotherapy was identified, and later gynecological oncology was defined as a separate area and a medical specialty. During this time women became more well-informed about gynecological diseases and different symptoms. Due to gradually increased access to medical services and specialists, women started to consult a doctor for various symptoms, resulting in earlier detection of CC.

The number of CC cases gradually increased until the 1950s and thereafter leveled out to quite a stable number until 1960. This could be ascribed to an increased population at risk and improved access to medical services leading to a rise in the diagnosis of CC.

Organized screening programs for detection of the precancerous stages of CC with the Papanicolaou method were ongoing in a few counties in Sweden in the late 1950s and early 1960s. This organized general CC screening progressed rapidly. In 1973, the whole female population in the recommended age group 30–49 years (except women in the city of Gothenberg) had been offered the test. The programs were later extended to cover women in the age group 23–59 years every third year 20–22.

During this 90-year period there has also been an obvious change in known risk factors and an increase in knowledge concerning the etiology of CC. There has been a change in smoking habits 23, and also a change in sexual behavior, and in the 1960s exogenous hormones and contraceptive pills were introduced.

Studies have shown that SCC and AC of the cervix share most risk factors, with the exception of smoking (for which the risk is elevated for SCC but not for AC) 24–26. As is the case for SCC, HPV appears to be the key risk factor for AC 27.

Studies have shown that the relative risk of CC and its precursor lesions is increased in current users of oral contraceptives and declines after use ceases 28, but the increase in risk does not seem to be associated with histology 29. It was observed that hormone replacement therapy, especially unopposed estrogens, was positively associated with ACs. Noncontraceptive hormones were negatively but weakly associated with SCCs 30.

Thus, the changing prevalence of oncogenic types of HPV since the 1960s may have contributed to the increase in AC. Moreover, increased use of unopposed estrogens as hormone replacement therapy in the 1980s and 1990s may have affected the risk for cervical AC as was the case for endometrial AC. Increased use of contraceptive pills since the 1960s may likewise have influenced the risk due to increased vulnerability to HPV infection caused by the migration of the transformation zone.

In summary, in this material comprising 90 years' experience with treating CC we have seen a change in the annual number of cases and in the distribution by age, stage, and histopathology. Some of these changes are mainly attributed to the introduction of screening programs but they are also related to social changes with increased availability of health care, and increased knowledge in medical science and concerning the etiology of CC. In addition, there has been a marked change in the risk factors for CC during this time period that may have affected the variables studied.
